# TUG1/miR-133b/CXCR4 axis regulates cisplatin resistance in human tongue squamous cell carcinoma

**DOI:** 10.1186/s12935-020-01224-9

**Published:** 2020-05-06

**Authors:** Ke Zhang, Hong Zhou, Bo Yan, Xuanping Cao

**Affiliations:** The First Affiliated Hospital of Zhengzhou University, No. 1 Jianshe East Road, Zhengzhou, 450000 Henan China

**Keywords:** TSCC, Cisplatin resistance, TUG1, miR-133b, CXCR4

## Abstract

**Background:**

Long noncoding RNA taurine upregulated 1 (TUG1) has been reported to play an important role in human cancers. However, little is known about the role of TUG1 in drug resistance and its mechanism in tongue squamous cell carcinoma (TSCC).

**Methods:**

Twenty-one cisplatin-sensitive or resistant TSCC patients were enrolled in this study. Cisplatin-resistant cells (SCC25/CDDP and CAL27/CDDP) were used for experiments in vitro. Transfection was performed using Lipofectamine 2000 transfection reagent. The levels of TUG1, microRNA-133b (miR-133b) and cysteine-X-cysteine chemokine receptor 4 (CXCR4) were measured by quantitative real-time polymerase chain reaction or western blot. The cisplatin resistance was investigated by cell viability, transwell invasion and apoptosis assays. The interactions among TUG1, miR-133b and CXCR4 were evaluated by luciferase reporter assay and RNA immunoprecipitation. Murine xenograft model was established using the stably transfected CAL27/CDDP cells.

**Results:**

TUG1 expression was elevated in cisplatin-resistant TSCC tissues and cells compared with that in sensitive group and its knockdown inhibited cisplatin resistance to SCC25/CDDP and CAL27/CDDP cells. miR-133b was targeted via TUG1 and its overexpression suppressed cisplatin resistance. Moreover, CXCR4 was a target of miR-133b. CXCR4 silence repressed cisplatin resistance, which was reversed by miR-133b knockdown. The level of CXCR4 protein was decreased by inhibition of TUG1 and recuperated by miR-133b knockdown. Besides, interference of TUG1 attenuated tumor growth by regulating miR-133b and CXCR4 in vivo.

**Conclusion:**

Downregulation of TUG1 impeded cisplatin resistance in TSCC-resistant cells by mediating miR-133b and CXCR4, indicating TUG1 as a promising target for TSCC chemotherapy.

## Background

Tongue and oral cavity are main sites of neoplasms or infections [1]. Oral cavity squamous cell carcinoma is one of the most common malignancies of human cancers [2]. Tongue squamous cell carcinoma (TSCC) is the main type of oral cavity squamous cell carcinoma with poor outcomes [3, 4]. Chemotherapy is an important strategy for TSCC treatment [5], while development of resistance limits its efficacy. Hence, much hope is placed on exploring novel avenues for reducing the drug resistance.

The networks of noncoding RNAs, including long noncoding RNAs (lncRNAs) and microRNAs (miRNAs), play important roles in the drug resistance in cancers [6]. LncRNAs without the open reading frame display multiple biological functions and regulate cellular processes, including cisplatin resistance [7]. In TSCC, lncRNA acts as an oncogene or tumor suppressor by functioning as competing endogenous RNA (ceRNA) [8]. For example, Wang et al. reporte that lncRNA urothelial cancer-associated 1 knockdown promotes cisplatin sensitivity to TSCC cells by regulating phosphatidylinositol 3-kinase (PI3K)/protein kinase B (Akt) pathway [9]. LncRNA taurine upregulated 1 (TUG1) has been suggested as an oncogenic lncRNA in many cancers [10]. More particularly, the available evidence indicates that TUG1 is aberrantly expressed in TSCC tissues [11]. However, the role of TUG1 in drug resistance and its mechanism remain poorly understood.

miRNAs are the small noncoding RNAs regulating mRNA expression through induction of RNA induced silencing complex (RISC) by binding their 3′ untranslated regions (3′ UTR), which are suggested to participate in the diagnosis and therapy of TSCC [12]. miR-133b, as a member of cononical muscle-specific miRNAs family, plays diverse roles in human diseases or cancers [13]. The previous study shows that miR-133b might be associated with TSCC progression [14]. Nevertheless, little is known about the biological function of miR-133b in TSCC. The former finding suggests that cysteine-X-cysteine chemokine receptor 4 (CXCR4) is dysregulated in TSCC and predicts poor outcomes of patients [15]. We hypothesized that miR-133b and CXCR4 might be involved in TUG1-driving cisplatin resistance. Hence, this research investigated the impact of TUG1 on cisplatin resistance and explored the potential network of TUG1/miR-133b/CXCR4 in vitro.

## Materials and methods

### Patients and specimens

Twenty-one cisplatin-sensitive or resistant TSCC patients who have received cisplatin-based treatment were recruited from the First Affiliated Hospital of Zhengzhou University via surgical resection, which was classified according to the response evaluation criteria [16]. Tumor samples were collected and immediately stored at − 80 °C until used. All participants involved in this study have provided the written informed consent, and the study protocols were permitted by the Ethics Committee of the First Affiliated Hospital of Zhengzhou University and performed in accordance with the Declaration of Helsinki.

### Cell culture and transfection

The TSCC cell lines (SCC25 and CAL27) were purchased from American Type Culture Collection (Manassas, VA, USA). The cisplatin resistant cell lines (SCC25/CDDP and CAL27/CDDP) were established by stimulating sensitive cells with escalating doses of cisplatin (Sigma, St. Louis, MO, USA) as previously reported [16]. All cells were cultivated in Dulbecco’s Modified Eagle Medium (Gibco, Carlsbad, CA, USA) containing 10% fetal bovine serum (Hyclone, Logan, UT, USA) at 37 °C and 5% CO_2_.

Small interfering RNA (siRNA) targeting TUG1 (si-TUG1), siRNA targeting CXCR4 (si-CXCR4), siRNA negative control (si-NC), pcDNA and pcDNA-TUG1 overexpression vector (TUG1) were synthesized by Genepharma (Shanghai, China). The miRNA mimic or inhibitor targeting miR-133b (miR-133b or in-miR-133b) and their corresponding negative control (miR-NC or in-miR-NC) were purchased from RIBOBIO (Guangzhou, China). These oligonucleotides or vectors were transfected into SCC25/CDDP and CAL27/CDDP cells using Lipofectamine 2000 transfection reagent (Invitrogen, Carlsbad, CA, USA). The cells were harvested at 24 h after the transfection, and the following experiments were conducted.

### Quantitative real-time polymerase chain reaction (qRT-PCR)

The tissues or cells were incubating with TRIzol reagent (Invitrogen) for total RNA extraction according to the manufacturer’s instructions. For analysis of lncRNA or mRNA expression, the RNA was reversely transcribed using M-MLV reverse transcriptase kit (Invitrogen). For detecting miRNA level, cDNA was synthesized using All-in-One™ miRNA First stand cDNA Synthesis kit (GeneCopoeia, Rockville, MD, USA). The qRT-PCR was performed using SYBR green (Applied Biosystems, Foster City, CA, USA) and the specific primers on ABI 7500fast system. Referred to as the threshold cycle (Ct), the qRT-PCR outcome indicates the cycle at which the florescence signal exceeded a defined background threshold [17]. The Ct values were provided from the qRT-PCR instrumentation. The relative expression levels of miR-133b, TUG1 and CXCR4 were detected with U6 small RNA or GAPDH as internal control via 2^−ΔΔCt^ method [18]. The specific primers for miR-133b or U6 were purchased from GeneCopoeia and primers for TUG1, CXCR4 or GAPDH were listed as follows: TUG1 (Forward, 5′-GACAGAGGCGACAGGAACGACG-3′; Reverse, 5′-CACCATGCAACATCGAACCG-3′), CXCR4 (Forward, 5′-ACTACACCGAGGAAATGGGCT-3′; Reverse, 5′-CCCACAATGCCAGTTAAGAAGA-3′), and GAPDH (Forward, 5′-ATTCCATGGCACCGTCAAGGCTGA-3′; Reverse, 5′-TTCTCCATGGTGGTGAAGACGCCA-3′).

### Cisplatin resistance analysis

The cisplatin resistance was investigated by cell viability, transwell invasion and apoptosis assays. For assay of cell viability, cells with or without treatment of cisplatin were seeded into 96-well plates at a density of 3, 000 cells per well. After the culture for 0, 24, 48, 72 or 96 h, cells were interacted with 10 μL reagent of cell counting kit-8 (CCK-8) (Beyotime, Shanghai, China) for another 2 h at 37 °C, followed by optical density measurement at 450 nm with a microplate reader (Bio-Rad, Hercules, CA, USA). The IC50 of cisplatin is the cisplatin concentration reducing viability by 50%.

For transwell invasion assay, the Matrigel (Becton–Dickinson, Franklin Lakes, NJ, USA) was used to coat the transwell chamber (24-well-plate format, Corning, Corning, NY, USA). And 200 μL cell suspension (2 × 10^5^ cells/mL) was seeded in the upper chambers and cultured at 37 °C for 24 h. The noninvasive cells were removed with a cotton swabs, and the invasive cells through the membranes were stained with 0.5% crystal violet (Sigma), observed and counted using a microscope (Olympus, Tokyo, Japan) with three random fields at 200× magnification.

For apoptosis analysis of flow cytometry, the transfected cells were incubated with an Annexin V-fluorescein isothiocyanate (FITC)/propidium iodide (PI) apoptosis detection kit (Yeasen, Shanghai, China) according to the manufacturer’s instructions. The apoptotic cells (Annexin V-FITC^+^ and PI^+/−^) were examined using a flow cytometer with BD FACSDiva™ software (Becton–Dickinson).

### Luciferase reporter assay

The potential binding sites of miR-133b and TUG1 or CXCR4 were predicted by mirtarBase or DIANA tools online. To explore the interaction between TUG1 and miR-133b, the wild type (WT) or mutant (MUT) luciferase reporter construct for TUG1 was generated by pGL3 vector (Promega, Madison, WI, USA) through cloning the sequences of TUG1 containing putative binding sites of miR-133b (5′-GGACCAA-3′) or mutant seed sites (5′-CCUGGUU-3′), named as TUG1 WT or TUG1 MUT, respectively. For exploring the relationship of miR-133b and CXCR4, the 3′ untranslated regions (3′-UTR) sequences of CXCR4 containing the putative or MUT binding sites of miR-133b were inserted in the downstream of luciferase gene in pGL3 vector, creating the luciferase reporter vector CXCR4 WT or CXCR4 MUT. SCC25/CDDP and CAL27/CDDP cells were co-transfected with luciferase reporter vectors, control vector and miR-133b mimic or miR-NC using Lipofectamine 2000 transfection reagent. After 48 h of transfection, the analysis of luciferase activity was performed using luciferase reporter assay kit (Promega).

### RNA immunoprecipitation (RIP)

For Ago2 RIP assay, SCC25/CDDP and CAL27/CDDP cells were transfected with miR-133b mimic or miR-NC, and the analysis was performed with a Magna RNA immunoprecipitation kit (Millipore, Billerica, MA, USA). In brief, after 48 h of transfection, cells were lysed in RIP buffer containing magnetic beads conjugated with Ago2 or IgG antibody. The immunoprecipitated RNAs were isolated by TRIzol reagent and the enrichment level of TUG1 was analyzed by qRT-PCR.

### Western blot

For protein extraction, cells or tissues were washed with cold PBS and lysed in RIPA lysis buffer (Beyotime). Total proteins were separated using SDS-PAGE, transferred to polyvinylidene difluoride membranes (Millipore) and blocked with 5% non-fat milk for 1 h at room temperature. Subsequently, the membranes were incubated with primary antibodies against CXCR4 (ab124824; Abcam, Cambridge, MA, USA) or β-actin (ab8227; Abcam) overnight at 4 °C, interacted with horseradish peroxidase-conjugated secondary antibody (ab6721; Abcam) for 2 h at room temperature and then visualized using enhanced chemiluminescence chromogenic substrate (Beyotime) and X-OMAT BT film (Carestream Health, Rochester, NY, USA). The gray values of CXCR4 and β-actin were analyzed using Image J software (NIH, Bethesda, MD, USA). The relative expression of CXCR4 was analyzed with β-actin as loading control and normalized to the control group.

### Murine xenograft assay

BALB/c nude mice (male, four-week-old) were purchased from Vital River Laboratory Animal Technology (Beijing, China) and randomly divided in two groups (n = 6 per group). The experiment was approved by the Animal Research Committee of the First Affiliated Hospital of Zhengzhou University and performed in accordance with the guidelines of the National Animal Care and Ethics Institution. CAL27/CDDP cells were transfected with the lentivirus harboring short hairpin RNA targeting TUG1 (sh-TUG1) or negative control (sh-NC) constructed by GeneCopoeia. The mice were infected subcutaneously with the stably transfected cells (5 × 10^6^), and tumor volumes were monitored every week and calculated with the formula: volume (mm^3^) = width^2^ × length/2. After 35 days following the inoculation, the mice were killed and tumor samples were weighed and used for further studies.

### Statistical analysis

The statistical analysis was carried out using GraphPad Prism 7 (GraphPad Inc., La Jolla, CA, USA). The data were presented as the mean ± standard deviation (S.D.) from three independent experiments. The difference between two groups was analyzed by Student’s *t*-test and that between multiple comparisons was performed by one-way analysis of variance (ANOVA) followed via Tukey test. *P *< 0.05 was regarded as statistically significant difference.

## Results

### The expression of TUG1 is enhanced in cisplatin-resistant TSCC tissues and cells

**Fig. 1:**
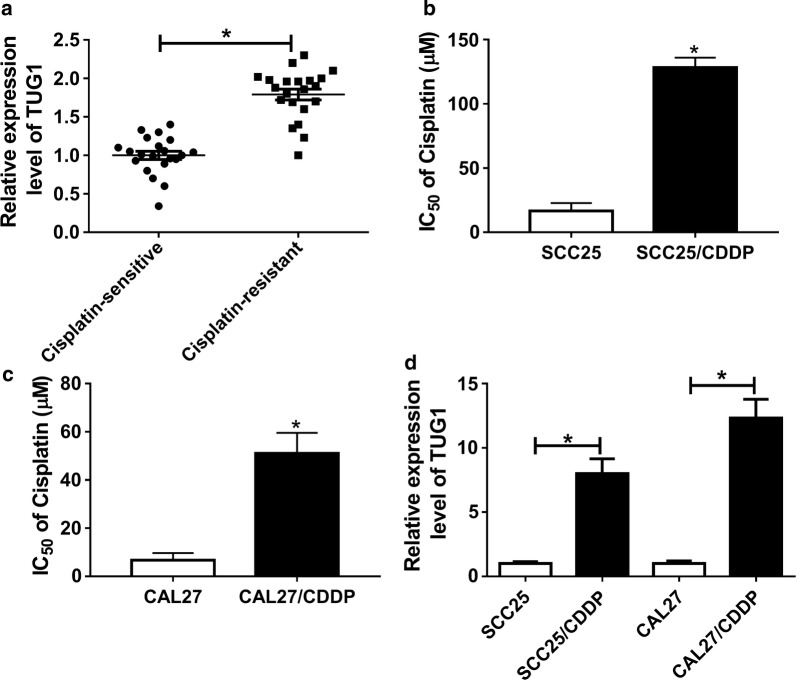
The expression of TUG1 in cisplatin-resistant TSCC tissues and cells. **a** The expression of TUG1 was measured in cisplatin-resistant and sensitive TSCC tissues by qRT-PCR. **b**, **c** The IC50 of cisplatin was analyzed in cisplatin-resistant and sensitive TSCC cells by CCK-8. **d** The level of TUG1 was detected in cisplatin-resistant and sensitive TSCC cells by qRT-PCR. *P < 0.05

To explore the potential role of TUG1 in TSCC, the expression of TUG1 was measured in TSCC patients with cisplatin treatment. The results showed that the level of TUG1 was significantly elevated in tissues from cisplatin-resistant patients compared with that in cisplatin-resistive group (Fig. 1a). Moreover, the cisplatin-resistant cells were established by TSCC cells, which were validated by the elevated IC50 of cisplatin in SCC25/CDDP and CAL27/CDDP cells after 48 h of cisplatin exposure (Fig. 1b, c). In addition, the expression of TUG1 was detected in cisplatin-resistant or sensitive cells, and the data displayed higher TUG1 level in the resistant cells than that in sensitive cells (Fig. 1d).

### Knockdown of TUG1 inhibits cisplatin resistance in cisplatin-resistant TSCC cells

**Fig. 2:**
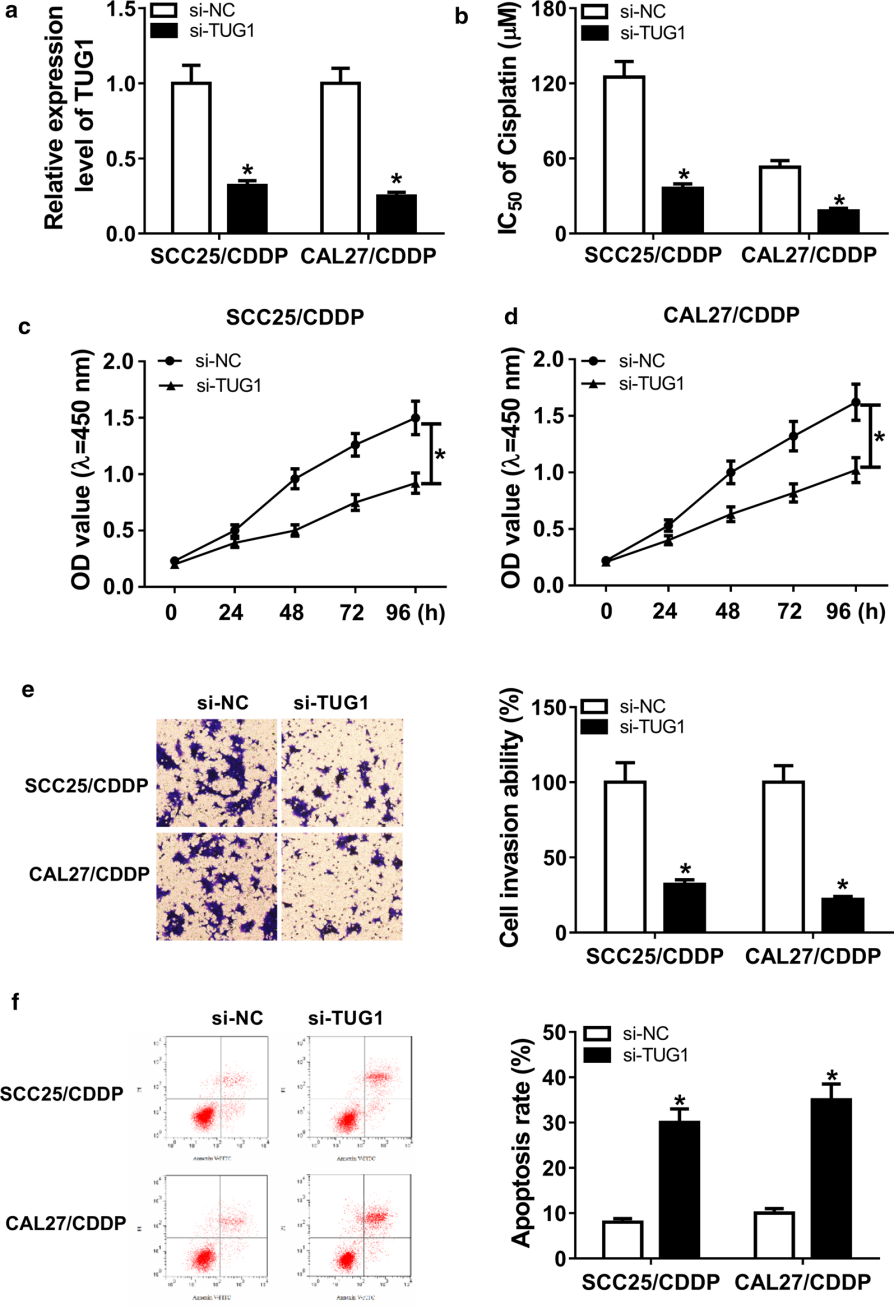
The effect of TUG1 knockdown on cisplatin resistance in cisplatin-resistant TSCC cells. Cisplatin-resistant TSCC cells were transfected with si-TUG1 or si-NC for 24 h. Then the abundance of TUG1 (**a**), IC50 of cisplatin (**b**), viability (**c**, **d**), invasion (**e**) and apoptosis (**f**) were measured by qRT-PCR, CCK-8, transwell or flow cytometry, respectively. *P < 0.05

In order to investigate the biological function of TUG1 on cisplatin resistance, SCC25/CDDP and CAL27/CDDP cells were transfected with si-TUG1 or si-NC. The transfection efficacy was identified by qRT-PCR with the results of down-regulation of TUG1 expression in cells transfected with si-TUG1 (Fig. 2a). After treatment of different concentrations of cisplatin for 48 h, the IC50 of cisplatin was remarkably decreased by knockdown of TUG1 in the two cisplatin-resistant cell lines (Fig. 2b). Moreover, interference of TUG1 notably suppressed the viability of SCC25/CDDP and CAL27/CDDP cells (Fig. 2c, d). Additionally, the data of transwell invasion assay revealed that silence of TUG1 significantly reduced the invasive ability of SCC25/CDDP and CAL27/CDDP cells (Fig. 2e). Besides, the results of flow cytometry showed that transfection of si-TUG1 remarkably promoted the resistant cell apoptosis (Fig. 2f).

### miR-133b is bound to TUG1

**Fig. 3:**
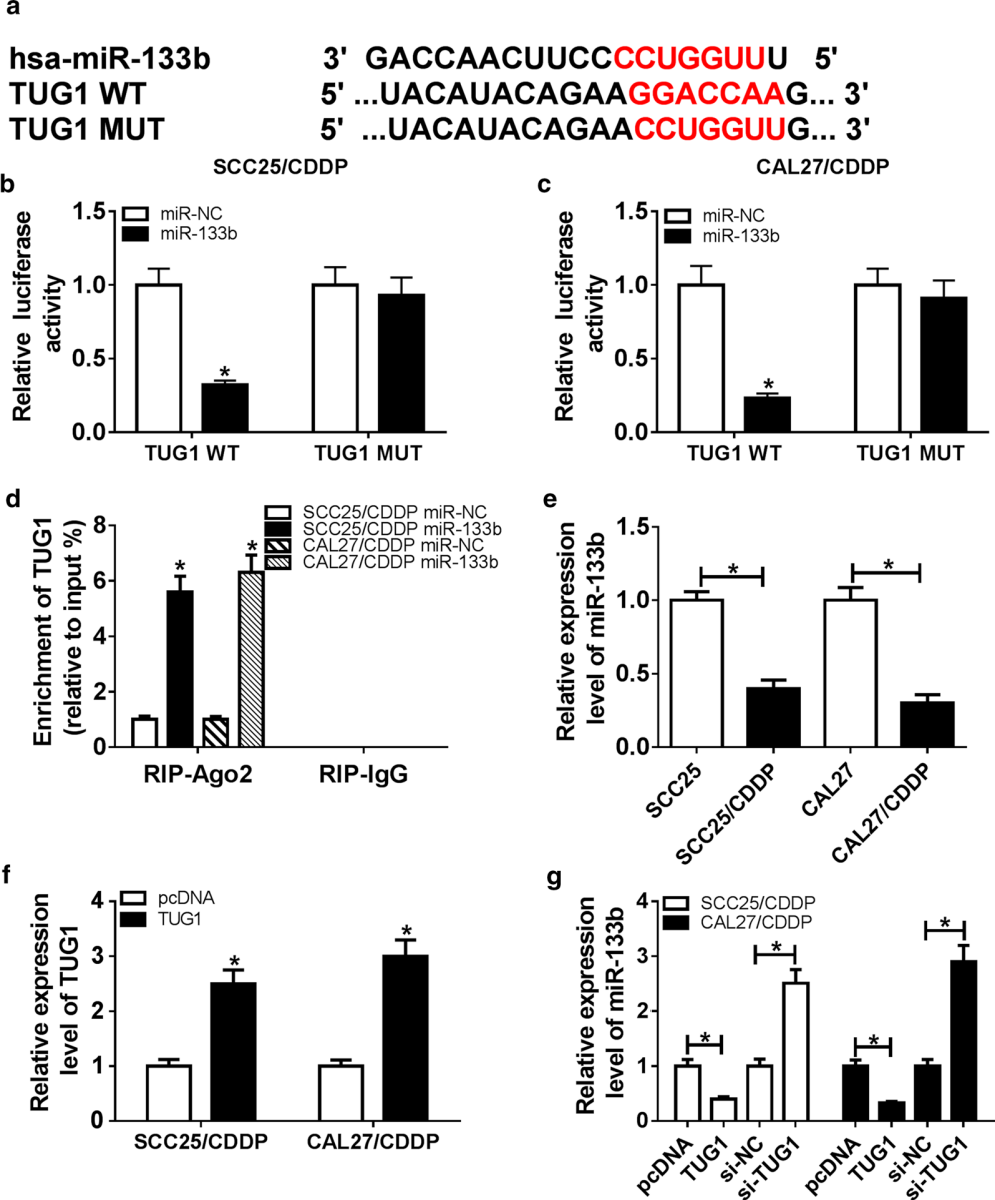
The association between miR-133b and TUG1. **a** The putative binding sites of TUG1 and miR-133b. **b**, **c** Luciferase activity was detected in SCC25/CDDP and CAL27/CDDP cells co-transfected with TUG1 WT or TUG1 MUT and miR-133b mimic or miR-NC. **d** The enrichment level of TUG1 was measured in SCC25/CDDP and CAL27/CDDP cells transfected with miR-133b mimic or miR-NC. **e**, **f** The expression of miR-133b was examined in cisplatin-resistant and sensitive TSCC cells by qRT-PCR. **g** The level of miR-133b was detected in SCC25/CDDP and CAL27/CDDP cells transfected with pcDNA, TUG1 overexpression vector, si-NC or si-TUG1. *P < 0.05

To elucidate the underlying mechanism allows TUG1 involved in cisplatin resistance, the potential miRNA target was explored by bioinformatics analysis. The putative binding sites of TUG1 and miR-133b were shown in Fig. 3a, suggesting that TUG1 might act as a decoy of miR-133b. To prove this prediction, we constructed luciferase reporter vectors TUG1 WT and TUG1 MUT, which was co-transfected into cells with miR-133b mimic or miR-NC. Overexpression of miR-133b significantly decreased the luciferase activity of SCC25/CDDP and CAL27/CDDP cells in TUG1 WT group, whereas it showed little impact on the activity in TUG1 MUT group (Fig. 3b, c). Moreover, RIP assay revealed that transfection of miR-133b mimic resulted in great enrichment of TUG1 in SCC25/CDDP and CAL27/CDDP cells in comparison to miR-NC group (Fig. 3d). Subsequently, the expression of miR-133b was measured in TSCC cells, and the results showed low expression of miR-133b in resistant cells compared with that in sensitive group (Fig. 3e). Furthermore, the effect of TUG1 on miR-133b expression was investigated in the two cell lines. As demonstrated in Fig. 3f, g, up-regulation of TUG1 induced by transfection of TUG1 overexpression vector led to obvious loss of miR-133b abundance, while its knockdown increased miR-133b level.

### TUG1 regulates cisplatin resistance in TSCC cells by sponging miR-133b

**Fig. 4:**
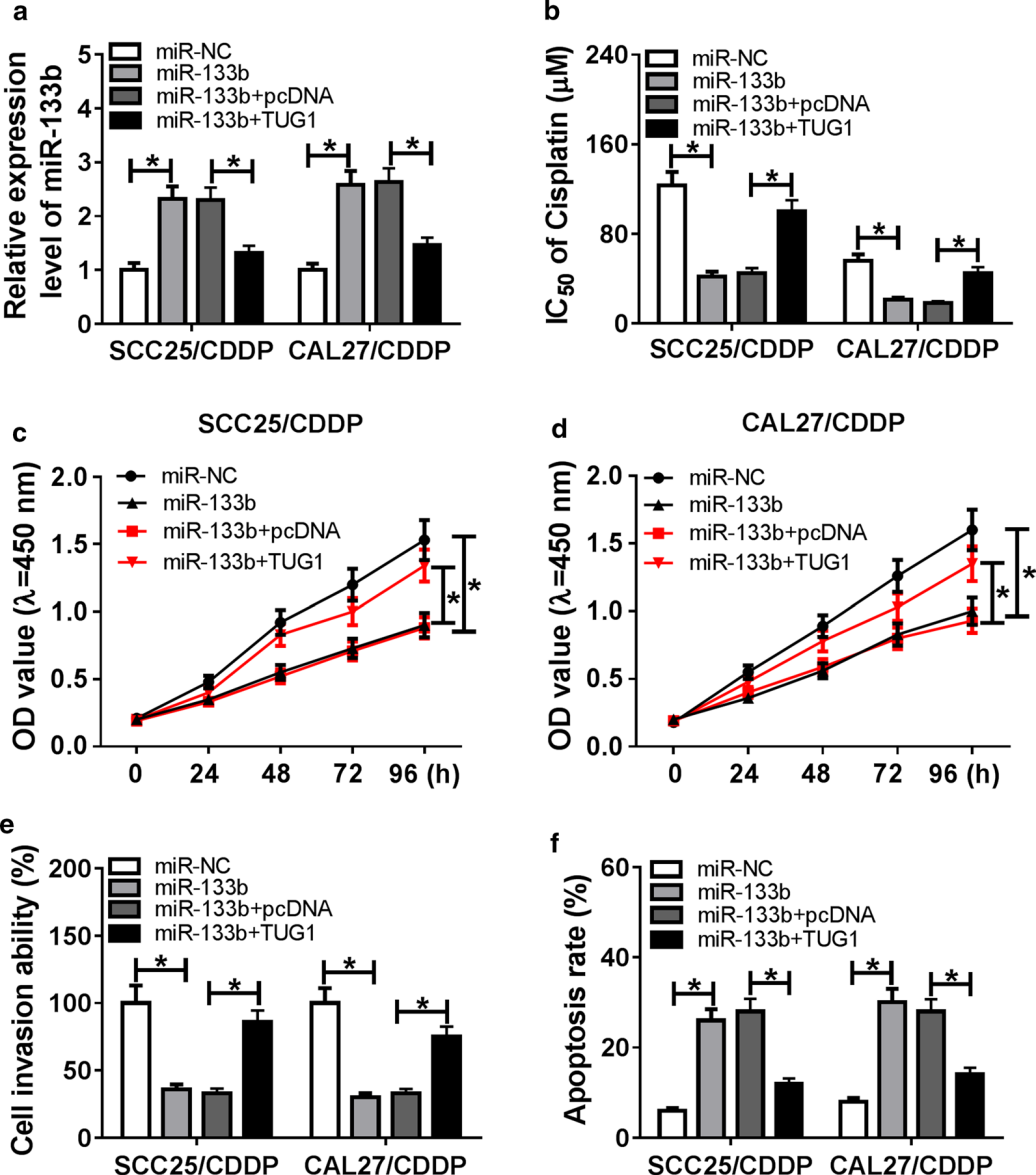
The regulatory effect of TUG1 on miR-133b-mediated cisplatin resistance in TSCC cells. The expression of miR-133b (**a**), IC50 of cisplatin (**b**), cell viability (**c**, **d**), invasion (**e**) and apoptosis (**f**) were detected in SCC25/CDDP and CAL27/CDDP cells transfected with miR-NC, miR-133b mimic, miR-133b mimic + pcDNA or TUG1 overexpression vector by qRT-PCR, CCK-8, transwell or flow cytometry, respectively. *P < 0.05

To validate whether miR-133b is required for TUG1-addressed cisplatin resistance, SCC25/CDDP and CAL27/CDDP cells were transfected with miR-NC, miR-133b mimic, miR-133b mimic + pcDNA or TUG1 overexpression vector. After 24 h of transfection, the abundance of miR-133b was abnormally enhanced by transfection of miR-133b mimic in the two cell lines, which was weakened by introduction of TUG1 overexpression vector (Fig. 4a). Moreover, the transfected cells were treated with different concentrations of cisplatin for 48 h, and results presented the IC50 of cisplatin was remarkably reduced by overexpression of miR-133b, which was alleviated by addition of TUG1 (Fig. 4b). Furthermore, the cell functional analyses described that accumulation of miR-133b induced inhibition of viability and invasive ability as well as promotion of apoptosis, while introduction of TUG1 counteracted this effect (Fig. 4c–f).

### CXCR4 is a target of miR-133b

**Fig. 5:**
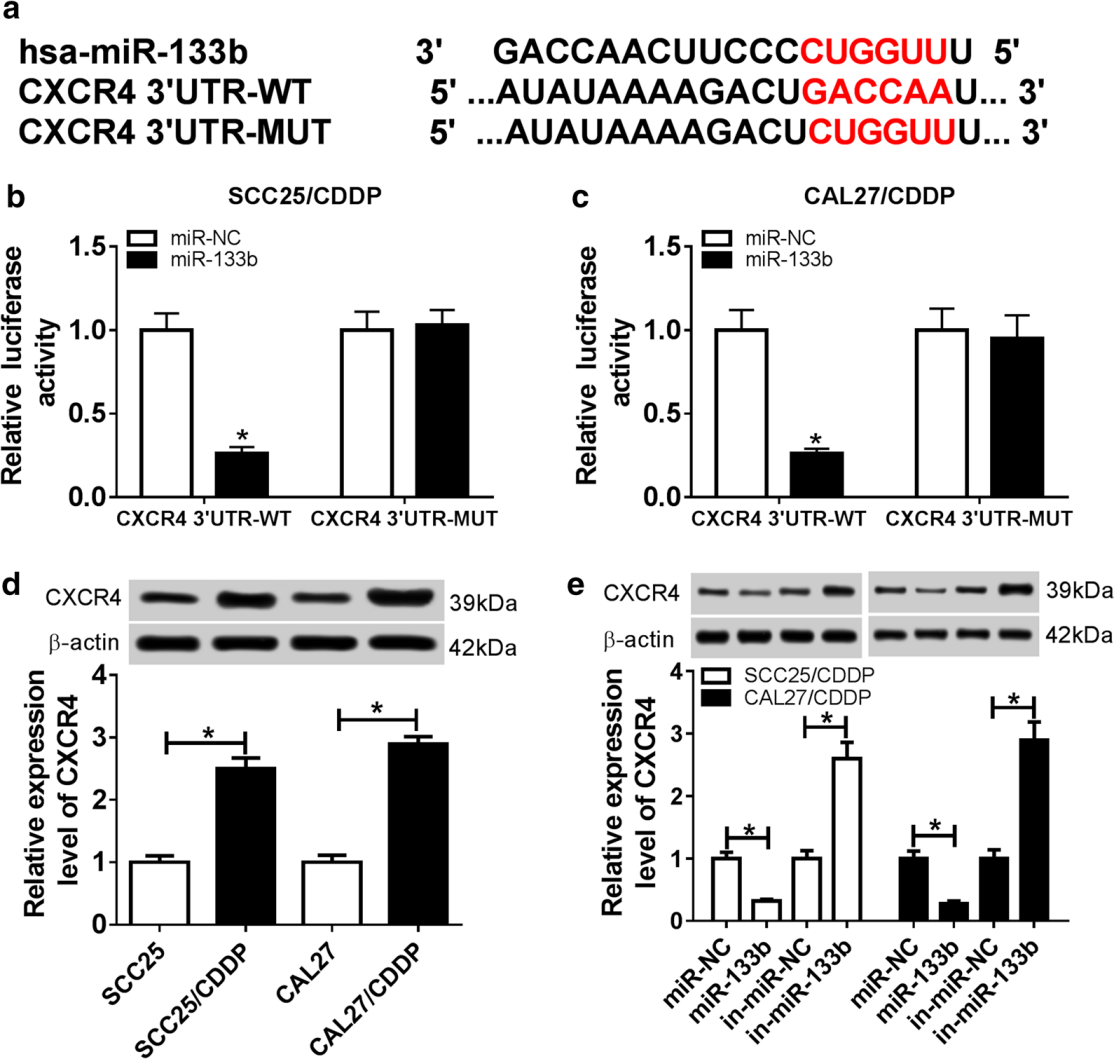
The relationship between CXCR4 and miR-133b. **a** The potential seed sites between miR-133b and CXCR4. **b**, **c** Luciferase activity was analyzed in SCC25/CDDP and CAL27/CDDP cells co-transfected with CXCR4 WT or CXCR4 MUT and miR-133b mimic or miR-NC. **d** The protein level of CXCR4 was detected in cisplatin-resistant and sensitive TSCC cells by western blot. **e** The protein abundance of CXCR4 was measured in SCC25/CDDP and CAL27/CDDP cells transfected with miR-NC, miR-133b mimic, in-miR-NC or in-miR-133b by western blot. *P < 0.05

To implicate the potential mechanism of miR-133b-drived cisplatin resistance, its target was explored, and the potential seed sites of miR-133b and CXCR4 were shown in Fig. 5a. Luciferase reporter assay was exploited to support this association, revealed by loss of luciferase activity in SCC25/CDDP and CAL27/CDDP cells co-transfected with CXCR4 WT and miR-133b mimic (Fig. 5b, c). Furthermore, the expression of CXCR4 protein was detected in TSCC cells, and cisplatin-resistant cells showed higher level of CXCR4 than sensitive cells (Fig. 5d). Meanwhile, the data of western blot displayed in Fig. 5e, that the abundance of CXCR4 was evidently inhibited by overexpression of miR-133b and up-regulated by knockdown of miR-133b.

### miR-133b mediates cisplatin resistance by targeting CXCR4

**Fig. 6:**
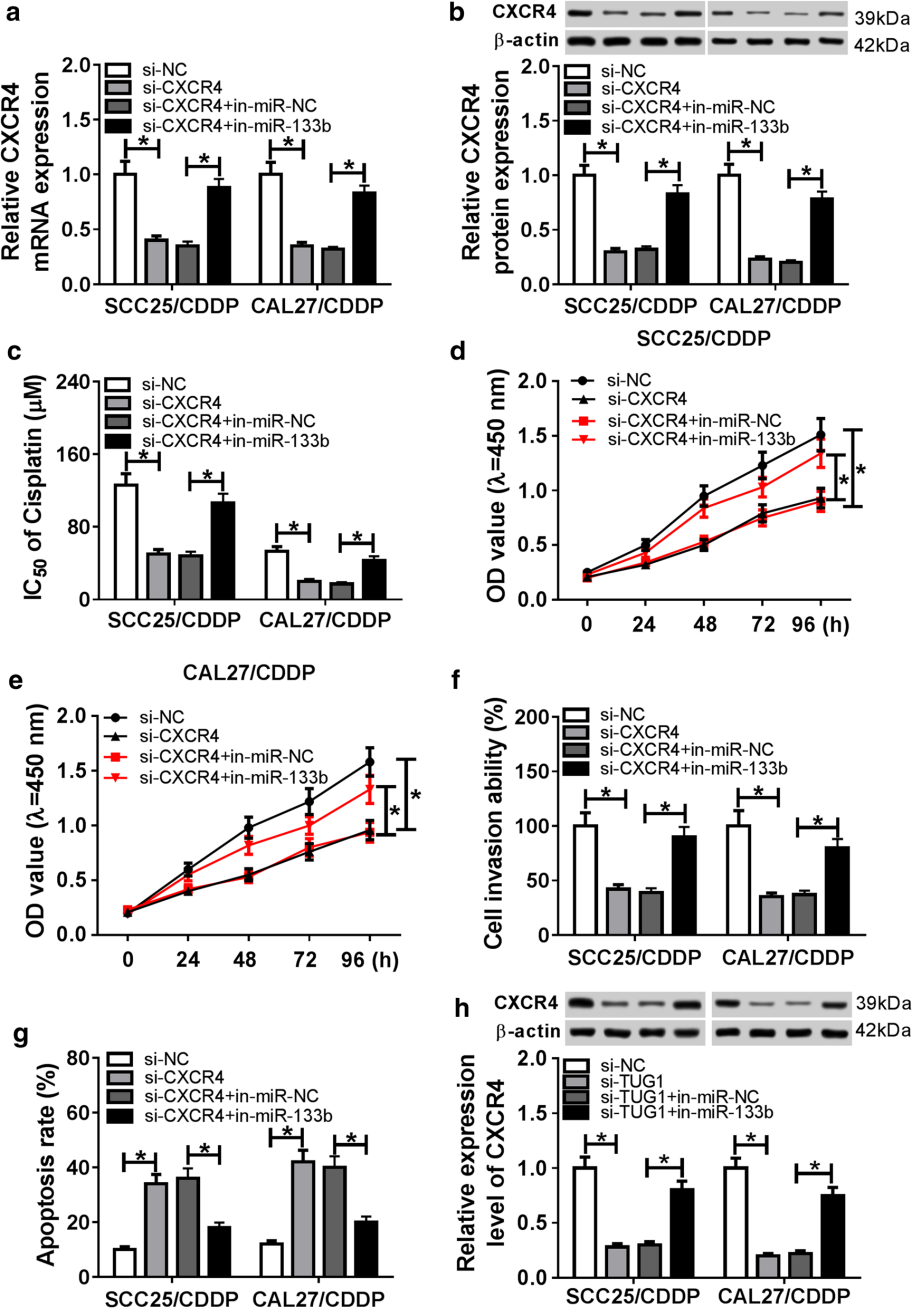
The regulatory effect of miR-133b on CXCR4-mediated cisplatin resistance of TSCC cells. The mRNA and protein levels of CXCR4 **a**, **b**, IC50 of cisplatin (**c**), cell viability **d**, **e**, invasion (**f**) and apoptosis (**g**) were measured in SCC25/CDDP and CAL27/CDDP cells transfected with si-NC, si-CXCR4, si-CXCR4 + in-miR-NC or in-miR-133b by qRT-PCR, western blot, CCK-8, transwell or flow cytometry, respectively. **h** The protein abundance of CXCR4 was detected in SCC25/CDDP and CAL27/CDDP cells transfected with si-NC, si-TUG1, si-TUG1 + in-miR-NC or in-miR-133b by western blot. *P < 0.05

To explore whether CXCR4 is involved in miR-133b-mediated cisplatin resistance, two resistant cell lines were transfected with si-NC, ssi-CXCR4, si-CXCR4 + in-miR-NC or in-miR-133b. As shown in Fig. 6a, b, the transfection efficacy was validated at transcriptional and protein levels. In addition, loss-of-function experiments demonstrated that silencing CXCR4 impeded cisplatin resistance, uncovered by inhibition of IC50 of cisplatin, cell viability and invasion as well as increase of apoptosis in SCC25/CDDP and CAL27/CDDP cells (Fig. 6c–g). However, knockdown of miR-133b attenuated the suppressive role of CXCR4 silence. Besides, the effect of TUG1 on CXCR4 expression was investigated at protein level. As displayed in Fig. 6h, interference of TUG1 decreased CXCR4 protein expression in the two cell lines, while miR-133b deficiency abrogated this impact.

### Interference of TUG1 attenuates CAL27/CDDP xenograft tumor growth by regulating miR-133b and CXCR4

**Fig. 7:**
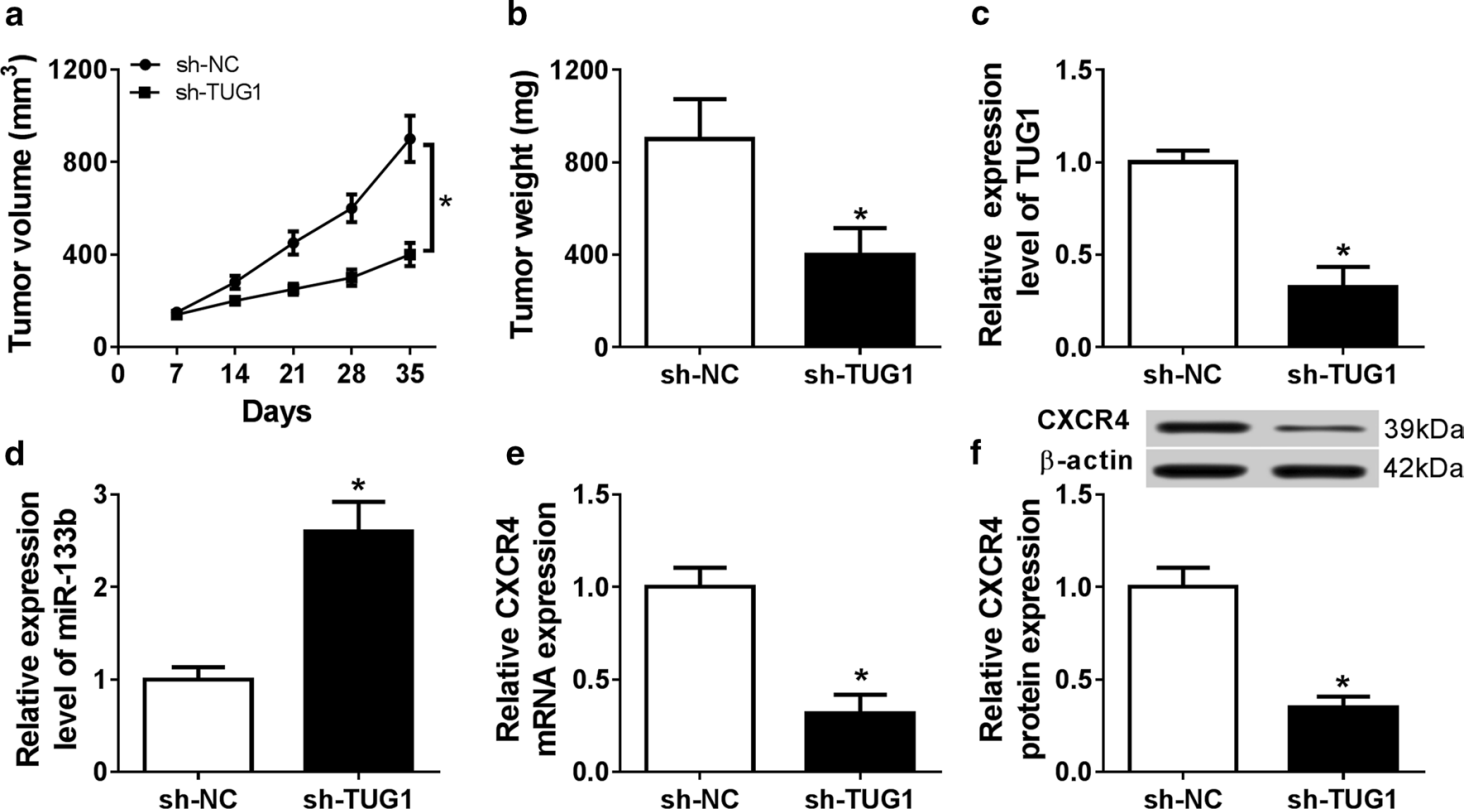
The effect of TUG1 interference on CAL27/CDDP xenograft tumor growth. **a** CAL27/CDDP xenograft tumor volume was measured every week. **b** Tumor weight was detected in each group at end point. The levels of TUG1 (**c**), miR-133b (**d**), CXCR4 mRNA and protein (**e**, **f**) were measured in two groups by qRT-PCR or western blot, respectively. *P < 0.05

To further analyze the impact of TUG1 on drug resistance of TSCC, CAL27/CDDP cells stably transfected with sh-TUG1 or sh-NC were used to establish the xenograft model in vivo. After 5 weeks following the cell inoculation, tumor volume and weight were greatly inhibited in sh-TUG1 group compared with those in sh-NC group (Fig. 7a, b). Moreover, molecular analyses were performed in the harvested tumor tissues. The expression of TUG1 was obviously decreased in sh-TUG1 group compared with that in sh-NC group (Fig. 7c). However, miR-133b level presented an opposite trend in the two groups (Fig. 7d). Additionally, the abundance of CXCR4 was significantly reduced at mRNA and protein levels in xenograft tissues induced by CAL27/CDDP cells with transfection of sh-TUG1 (Fig. 7e, f).

## Discussion

Chemoresistance blocks the efficacy of chemotherapy in human cancers. The previous work demonstrated that lncRNA could correlate with the metastasis, drug resistance along with clinical outcome in human cancers [19]. In the present study, we provided the first view on the sensitized role of TUG1 down-regulation to cisplatin in TSCC and first elucidated the ceRNA network of TUG1/miR-133b/CXCR4.

Xu et al. reported that TUG1 was up-regulated and contributed to cisplatin resistance through regulating programmed cell death protein 4 (PDCD4) by enhancer of zeste homolog 2 (EZH2) in esophageal squamous cell carcinoma [20]. However, Tang et al. showed that TUG1 was down-regulated and its overexpression enhanced cisplatin sensitivity by sponging miR-197 in triple negative breast cancer [21]. These findings revealed the association between TUG1 and cisplatin resistance, but the inconsistent impacts might be caused because of the different microenvironment in varying cancers. The report of Li et al. displayed high expression of TUG1 in TSCC tissues compared with that in normal samples, and it might promote TSCC progression [11]. However, there is no direct evidence in support of TUG1-addressed cisplatin resistance in TSCC. In this study, we found that TUG1 expression was increased in cisplatin-resistant tissues or cells compared with that in sensitive groups, suggesting that TUG1 might facilitate cisplatin resistance in TSCC. Subsequently, loss-of-function experiments revealed that TUG1 knockdown inhibited IC50 of cisplatin, cell viability and invasion and increased apoptosis, indicating the suppressive role of TUG1 knockdown in cisplatin resistance in TSCC. However, how TUG1 addresses cisplatin resistance remains elusive. The emerging evidence suggested that TUG1 could affect the drug resistance by functioning as a ceRNA of miR-186 in colorectal cancer [22]. Apart from this, Zhang et al. also provided a ceRNA network of TUG1 by interacting with miR-133a [23]. miR-133b has the homologous cluster with miR-133a, predicting they might play same roles by similar pathway. Here we first validated the interaction between TUG1 and miR-133b in TSCC cells, indicating the potential role of miR-133b in TUG1-drived process of TSCC.

Former effort suggested that miR-133b expression was decreased in TSCC cells [24], and we further provided lower expression of miR-133b in resistant cells than that in sensitive cells. Furthermore, the gain-of-function experiments uncovered that miR-133b overexpression inhibited cisplatin resistance, suggesting that miR-133b might act as a sensitizer of cisplatin in TSCC, which is also in agreement with that in ovarian cancer or lung cancer cells [25, 26]. Although it was reported to play an opposite effect in hepatocellular carcinoma or osteosarcoma [27, 28], we hypothesized the difference might be induced the alteration of tumor microenvironment. In addition, our data showed that introduction of TUG1 overturned the impact of miR-133b, implicating that TUG1 addressed cisplatin resistance by sponging miR-133b. The ceRNA hypothesis suggests that promising mRNA is required for lncRNA-mediated network. This study confirmed the relationship of miR-133b and CXCR4 in TSCC cells, which was reported in colorectal cancer by previous study [29].

CXCR4 was suggested to be expressed in TSCC tissues [30]. Moreover, one emerging work showed that CXCR4 was up-regulated in TSCC-resistant cells and it promoted cisplatin resistance of TSCC [31]. Similarly, this paper also exhibited high expression of CXCR4 in resistant cells and showed that its silence suppressed cisplatin resistance of TSCC, which was also consistent with that in other cancers [32, 33]. Moreover, deficiency of miR-133b attenuated the effect of CXCR4 interference, suggesting the importance of CXCR4 for miR-133b-mediated resistance in TSCC. Besides, CXCR4 protein level was decreased by TUG1 knockdown and rescued by depletion of miR-133b, which supported that TUG1 might act as a ceRNA of miR-133b to regulate CXCR4 expression in TSCC cells. In vivo experiments also reflected the potential role of TUG1 in TSCC and the ceRNA network. This study focused on the role of TUG1 in drug resistance of TSCC, but its effect on TSCC progression is also poorly understood. More details about the impact of TUG1 on cellular processes in TSCC should be explored in future.

## Conclusion

Our results revealed that TUG1 knockdown inhibited cisplatin resistance in TSCC, possibly via regulating CXCR4 by sponging miR-133b. This study provides new theoretical basis for TUG1 in TSCC and indicates TUG1 as a promising target for chemotherapy.

## Data Availability

All data generated or analyzed during this study are included in this published article.

## Abbreviations

TUG1: Taurine upregulated 1
TSCC: Tongue squamous cell carcinoma
PI3K: Phosphatidylinositol 3-kinase
RISC: RNA induced silencing complex
CXCR4: Cysteine-X-cysteine chemokine receptor 4
CCK-8: Counting kit-8
